# NAD(P)H Drives the Ascorbate–Glutathione Cycle and Abundance of Catalase in Developing Beech Seeds Differently in Embryonic Axes and Cotyledons

**DOI:** 10.3390/antiox10122021

**Published:** 2021-12-20

**Authors:** Ewa Marzena Kalemba, Shirin Alipour, Natalia Wojciechowska

**Affiliations:** 1 Institute of Dendrology, Polish Academy of Sciences, Parkowa 5, 62-035 Kórnik, Poland; salipour@man.poznan.pl (S.A.); natalia.wojciechowska@amu.edu.pl (N.W.); 2 Department of General Botany, Institute of Experimental Biology, Faculty of Biology, Adam Mickiewicz University, Uniwersytetu Poznańskiego 6, 61-614 Poznań, Poland

**Keywords:** antioxidants, catalase, *Fagus sylvatica*, pyridine nucleotides, redox status, seed maturation

## Abstract

European beech is an important component of European lowland forests in terms of ecology, and produces irregular seeds categorized as intermediate due to their limited longevity. Removal of the excess of reactive oxygen species is crucial for redox homeostasis in growing plant tissues. Hydrogen peroxide (H_2_O_2_) is detoxified via the plant-specific ascorbate-glutathione cycle, and enzymatically, mainly by catalase (CAT). The reduced and oxidized (redox) forms of ascorbate (AsA, DHA) and glutathione (GSH, GSSG) decreased during maturation as the content of redox forms of nicotinamide adenine dinucleotide (NADH, NAD^+^) phosphate (NADPH, NADP^+^), cofactors of ascorbate–glutathione enzymes, declined and limited this cycle. The degree of oxidation of glutathione peaked at approximately 80%, at the exact time when the NADP content was the lowest and the NADPH/NADP^+^ ratio reached the highest values. The glutathione pool was reflected in changes in the NADP pool, both in embryonic axes (R^2^ = 0.61) and in cotyledons (R^2^ = 0.98). A large excess of NADPH was reported in embryonic axes, whereas cotyledons displayed more unified levels of NADP redox forms. As a result, anabolic redox charge and reducing power were higher in embryonic axes. CAT was recognized as two proteins, and the abundance of the 55 kDa protein was correlated with all redox forms of ascorbate, glutathione, NAD, and NADP, whereas the 37 kDa protein was oppositely regulated in embryonic axes and cotyledons. Here, we discuss the role of NAD(P) in the regulation of the ascorbate–glutathione cycle, catalase, and seed longevity concerning a putative role of NAD(P)H as a redox biomarker involved in predefining seed quality, because NAD(P)H-derived redox homeostasis was found to be better controlled in embryonic axes than cotyledons.

## 1. Introduction

European beech (*Fagus sylvatica* L.) is a deciduous tree, capable of reaching heights of up to 50 m, that is widespread across Europe and an important component of lowland forests in terms of ecology. Reproduction via seeds is complicated in this species. Beech trees begin to flower at 40–50 years old [1]. The mast crop occurs irregularly, usually at 10-year intervals [2]. Extreme interannual variation in seed production is caused by deficiencies in pollen synthesis [3]. Additionally, weather strongly affects the temporal patterns of masting events of beech [4]. The whole beech tree, especially in the case of seedlings, is sensitive to global warming, because its growth is strongly reduced by hot summers and a dry and cold first half of the vegetation period [5]. Additionally, the growth and quality of beech saplings, which enable natural regeneration, depend on the seed source, planting site, and local climate and ungulate browsing intensities [6]. The European Environment Agency states that Europe has been warming much faster than the global average. In this context, the reproductive success of beech is questionable because mast seeding became less effective [7]. Forestation practices are mainly based on long-term-stored beech seeds deposited in genebanks. Despite the acquisition of desiccation tolerance in developing seeds [8], beech seeds exhibit poor longevity during storage under typical conditions, and therefore are classified as an intermediate category between orthodox (desiccation tolerant) and recalcitrant (desiccation sensitive) seeds [9].

According to Goldberg et al. [10], embryo development comprises two stages, morphogenesis and maturation; and the latter has two distinct phases, early maturation, also known as the seed filling phase, and late maturation, when desiccation occurs [11,12]. Seed maturation events, particularly at the seed filling stage, predefine seed longevity [13,14]. Seed filling is a crucial growth stage accompanied by carbohydrate, protein, and lipids synthesis accumulation in seeds impacting seed quality [15], which declines when environmental drought and heat stress occur [16]. More precisely, overoxidation of macromolecules was found to be correlated with reduced seed longevity [17]. Recently, thiol metabolism in developing beech seeds was found to affect their storage capacity [18], indicating that the redox regulatory network modifies the control of metabolic and developmental plant processes [19]. Controlled oxidation is a well-known mechanism of the regulation of plant growth and development [20,21], including seed development [22], dehydration [23,24], and germination [25,26]. Cellular redox homeostasis is needed during organ formation, comprising a synchronized sequence of events enabling cell cycle progression, cell proliferation, cell differentiation, and organ architecture formation to attain physiological maturity and function [14,27].

The plant enzymatic and nonenzymatic antioxidant system is a multielement network enabling defense against oxidative stress [28]. Except for the major antioxidant enzyme catalase (CAT), the plant-specific ascorbate–glutathione cycle, which involves efficient scavenging of reactive oxygen species (ROS), predominantly hydrogen peroxide (H_2_O_2_), seems to play a principal role in redox regulation [29,30]. Importantly, the redox status of glutathione is strongly correlated with cellular levels of H_2_O_2_ [30]. The ascorbate–glutathione cycle is the central mechanism mitigating oxidative damage, particularly under stress conditions [31]. The cycle is also involved in the regulation of plant growth and development, and the predominant role is assigned to glutathione, which has been linked to seed development [32,33], germination [20,34,35], and longevity [36,37]. Seed priming with AsA and/or GSH was found to successfully diminish aging damage in seeds [38]. Ascorbate (Asc) is less studied than its cycle partner, but its roles beyond being an antioxidant molecule have been documented [39,40]. Studies involving Arabidopsis ascorbate and glutathione deficient mutants revealed that AsA and GSH might display different functions in stress tolerance, depending on stress-inducing agents and stress levels [41]. Switching between the redox forms of Asc (reduced form: ascorbic acid, AsA; oxidized form: dehydroascorbate, DHA) and glutathione (reduced form: glutathione, GSH; oxidized form: glutathione disulfide, GSSG) depends on the activity of enzymes, which is limited by the availability of their cofactors—the reduced forms of nicotinamide adenine dinucleotide (NADH) phosphate (NADPH). The oxidized form of NAD (NAD^+^) is synthesized and then phosphorylated to NADP, and both nucleotides undergo switches of redox forms, acting in metabolism and as signaling molecules in plants [42,43,44,45].

Redox signaling, combined with plant hormones, controls plant growth [27,46]. Redox balance is achieved differentially in developing orthodox and recalcitrant seeds [22], but little is known about this in intermediate seeds. Importantly, the NAD(P) content and redox status contribute to the desiccation tolerance of seeds [23], further affecting NAD(P)H-dependent reactions, including the ascorbate–glutathione cycle [24,25]. These data encouraged us to investigate whether NAD(P) modulates the ascorbate–glutathione cycle, especially when a method for the determination of redox forms of ascorbate, glutathione, and NAD(P) can be applied to the same sample [47]. Additionally, NAD^+^ accumulation is characteristic of orthodox tissues [23,48]. Therefore, developing beech seeds displaying this intermediate status are interesting to investigate in terms of whether redox homeostasis in this species is closer to the orthodox or recalcitrant phenotype. The availability of the reduced nicotinamide adenine dinucleotides is a limiting factor for the function of the ascorbate–glutathione cycle. Moreover, their content and redox state are related to the abundance of CAT. In this context, we hypothesize that pyridine nucleotides might be involved in the nonorthodox characteristics of beech seeds, further linking redox imbalance to reduced longevity. In contrast to cotyledons, embryonic axes of developing seeds are enriched with proteins ensuring redox homeostasis and response to stress [49]. Therefore, NAD(P)H should be considered in terms of possible involvement in determination of seed quality as a redox biomarker.

## 2. Materials and Methods

### 2.1. Plant Material

The experimental material consisted of beech seeds (*Fagus sylvatica* L.) collected at 13–18 weeks after flowering (WAF) from one single tree growing in the Kórnik Arboretum in Poland (52°15′ N 17°06′ E). At each harvest, the moisture content and dry mass were determined using three samples of 20 embryonic axes and 10 cotyledons by heating seed samples at 105 °C for 24 h. Seeds were extracted from the pericarp and seed coats, and samples containing 10 embryonic axes and 5 cotyledons were weighed, frozen in liquid nitrogen, and then stored at −80 °C until use.

### 2.2. Protein Extraction, Electrophoresis and Western Blot Analysis

Seed samples were ground to dry powder in liquid nitrogen. Proteins were extracted using a buffer consisting of Tris-Cl, glycerol, β-mercaptoethanol, and polyvinylpolypyrrolidone at 4 °C for 1 h, with vortexing every 15 min. After centrifugation for 20 min at 20,000× *g* at 4 °C, the amount of proteins was quantified using the Bradford [50] method. Electrophoresis (SDS-PAGE) was performed on 12% polyacrylamide gels. Twenty µg of protein samples were loaded in each lane (Figure S1). The protocol for Western blot experiment was carried out as described previously [24]. Proteins were transferred to the polyvinylidene fluoride (PVDF) membrane using Trans-Blot^®®^ Turbo™ (Bio-Rad, Hercules, CA, USA). Then, PVDF membrane was blocked in 5% skimmed milk (SM) dissolved in phosphate-buffered saline (PBS) at pH 7.4 for 1 h at RT. The primary antibodies raised against ascorbate peroxidase (catalog no.AS06 180, Agrisera, Sweden) and catalase (catalog no. AS09 501, Agrisera, Sweden) were diluted as recommended by the manufacturer (1:1000) in 5% SM/PBS. Secondary antibodies conjugated with horseradish peroxidase (HRP, catalog no. AS09 602, Agrisera, Sweden) were diluted 1:10,000 in 5% SM/PBS. To visualize protein bands, Clarity Western ECL substrate chemiluminescent detection reagent (Bio-Rad, Hercules, CA, USA) was used. Images were captured in a G:BOX Chemi XR5 instrument (Syngene, Cambridge, UK) to analyze detected bands densitometrically with the use of the UviBand (UviTec, Cambridge, UK) program. The density of bands (volume, V) was quantified as the sum of all 3D intensities (I) coded on a level gray scale ranging from 0 to 255. The band intensity was expressed in relative units calculated from V = Σn_i_I and the number of pixels within the band.

### 2.3. Determination of the Contents of Redox Forms

To determine the contents of the redox forms of ascorbate, glutathione, and NAD(P), the extract was prepared in accordance with a procedure described by Queval and Noctor [47]. Samples were homogenized in 0.2 M HCl and centrifuged for 10 min at 4 °C and 14,000 rpm. The homogenate was separated in two samples. In the first one, the pH was adjusted to 4.5–5 to measure Asc and glutathione. The second one was heated for 2 min at 100 °C, and after cooling, the pH was adjusted to 6–7 to measure NAD(P).The reactions were measured using an Infinite M200 PRO plate reader (Tecan, Männedorf, Switzerland) and Magellan software.

#### 2.3.1. Determination of Ascorbate

A combination of methods described by Hewitt and Dickes [51] and Queval and Noctor [47] was used for ascorbate determination. Total ascorbate (Asc; AsA + DHA) was assessed by converting the whole Asc extract to the reduced form by using 25 mM dithiothreitol at pH 4.7. AsA was measured in neutralized extracts by monitoring its absorption at 265 nm. The reaction mixture consisted of 0.1 mM acetic acetate buffer and 5 mM ethylenediaminetetraacetic acid (EDTA). The DHA was determined by subtracting free AsA from the total Asc. The concentrations were calculated on the basis of calibration curves prepared using AsA (Sigma-Aldrich, St. Louis, MO, USA) as a standard.

#### 2.3.2. Determination of Glutathione

The reduced and oxidized forms were determined according to the method of Griffith [52] adapted for use in a microplate reader [47]. The neutralized extract was treated with 2-vinylpyridine (2-VP) for 30 min at room temperature (RT) and centrifuged twice for 15 min at 4 °C and 14,000 rpm. The reaction mixture contained 120 mM NaH_2_PO_4_/10 mM EDTA, pH 7.5, 12 mM 5.5′-dithiobis(2-nitrobenzoic) acid (DTNB), 10 mM NADPH, MQ water, and extract (to measure total glutathione, GSH + GSSG) or 2-VP-treated extract (to determine the oxidized form GSSG), and glutathione reductase (0.2 U). The measurements were performed at 412 nm. The calculations were based on calibration curves prepared using GSH and GSSG (Sigma-Aldrich, St. Louis, MO, USA) as standards. The half-cell reduction potential of glutathione (*E*_GSSG/2GSH_) was calculated using the Nernst equation: *E*_GSSG/2GSH_ = *E*_0_ − (RT/nF)log([red]/[ox]). *E*_0_ = −240 mV (at pH 7); R, gas constant (8.314 JK^−1^ mol^−1^); T, temperature (K); n, number of electrons involved in the reaction; F, Faraday constant (9.6485 104C mol^−1^); red, molar concentration of GSH; ox, molar concentration of GSSG. *E*_0_ was adjusted to *E*_pH_ as described by Schafer and Buettner [53].

#### 2.3.3. Determination of NAD(P)

NAD(P) forms were measured using the method of Monéger et al. [54], in which reduced and oxidized forms were differentiated based on their ability to destruct in acid or base. To measure NAD(P)^+^, samples prepared as described in Section 2.3 were used, whereas to measure NAD(P)H, samples were additionally extracted in 0.2 M NaOH and neutralized with 0.2 N HCl. The reaction mixture contained 10 mM HEPES/2 mM EDTA (pH 7.5), 1.2 mM 2,6-dichlorophenolindophenol, 10 mM phenazine methosulfate, and neutralized extracts. The measurement of NAD was based on the conversion of ethanol via alcohol dehydrogenase, whereas the measurement of NADP was based on the conversion of glucose-6-phosphate via glucose-6-phosphate dehydrogenase. Reaction kinetics were measured at 600 nm. Concentration of the reduced and oxidized forms of NAD(P) was calculated on the basis of calibration curves prepared using reduced and oxidized forms of NAD and NADP (Sigma-Aldrich, St. Louis, MO, USA), respectively, as standards.

#### 2.3.4. Determination of NAD(P)-Originated Physiological Indices

The anabolic redox charge (ARC) and catabolic redox charge (CRC) were calculated using the equation described in Lorenc-Plucińska and Karolewski [55]: ARC = NADPH/(NADPH + NADP^+^), CRC = NADH/(NADH + NAD^+^). The reduction power was calculated using the equation (NADH + NADPH)/(NAD^+^ + NADH) + (NADP^+^ + NADPH) described by Quebedeaux [56], and was expressed as a ratio in the range of 0–1. The depth of dormancy was expressed as the NAD/NADP ratio according to Hunt et al. [57]. Phosphorylation capacity of NADK1 and NADK3 was calculated as product-to-substrate ratios NADP^+^/NAD^+^ and NADPH/NADH, respectively.

### 2.4. Statistical Analyses

The data were obtained from analyses of three independent biological replicates and three technical replicates each. Statistically significant differences are marked with different letters (ANOVA and Tukey’s test at *p* > 0.05). Proportional data were arcsine transformed prior to analysis using R statistical software to calculate Pearson’s correlation coefficients [58]. The corrplot package was used to construct correlation matrices [59].

## 3. Results

### 3.1. Ascorbate

**Figure 1:**
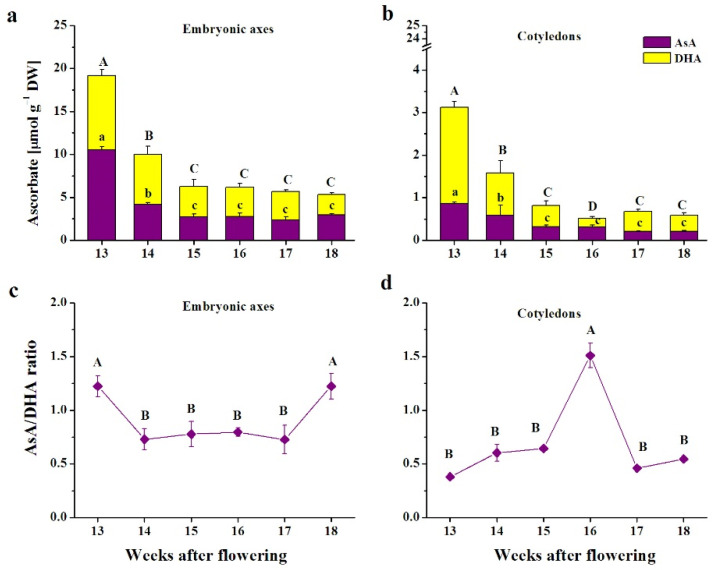
Levels of the reduced form (ascorbic acid, AsA) and oxidized (dehydroascorbate, DHA) form of ascorbate (**a**,**b**) and the AsA/DHA ratio (**c**,**d**) in embryonic axes (**a**,**c**) and cotyledons (**b**,**d**) of developing beech seeds. Data are shown as the means ± standard deviation (*n* = 3). Different letters indicate the statistical significance (one-way ANOVA, followed by Tukey’s test at *p* < 0.05). The capital letters refer to DHA (**a**,**b**).

The pattern of changes in Asc was identical in embryonic axes and cotyledons, while embryonic axes contained 10 times higher Asc levels (Figure 1). Beginning at the 15th WAF, Asc levels were unified until the end of maturation in both seed tissues, being 3 and 5 times lower in the embryonic axes and cotyledons, respectively, than at the initial maturation stage. DHA, the oxidized form of AsA, was the predominant form of Asc except in a few stages, in which the AsA/DHA ratio exceeded 1 (Figure 1c). The AsA/DHA ratio was lower in cotyledons except at the 16th WAF stage, when the ratio was tripled, and peaked at 1.5 (Figure 1d).

### 3.2. Glutathione

**Figure 2:**
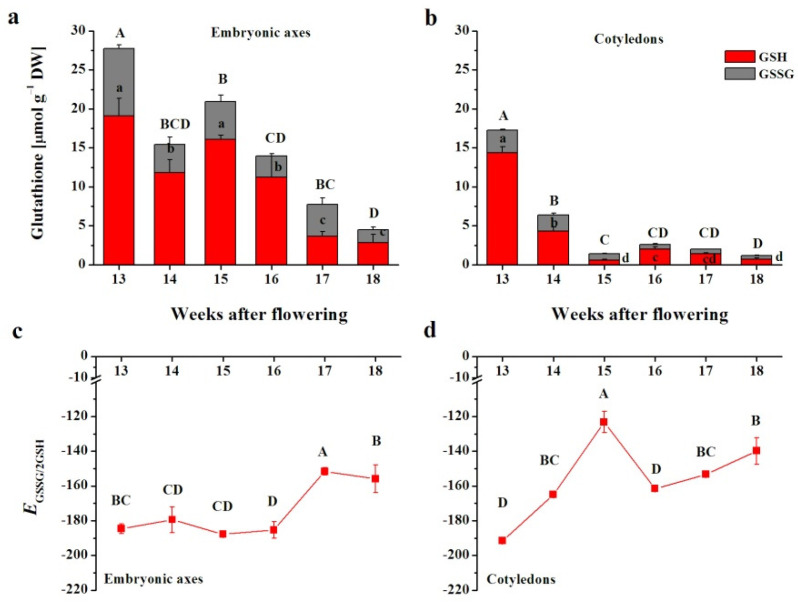
Levels of the reduced form of glutathione (GSH) and its oxidized form *glutathione* disulfide (GSSG) (**a**,**b**) and the half-cell reduction potential of glutathione (*E*_GSSG/2GSH_) (**c**,**d**) in embryonic axes (**a**,**c**) and cotyledons (**b**,**d**) of developing beech seeds. Data are the means of three independent replicates ± the standard error. Statistically significant differences are indicated with different letters (one-way ANOVA, followed by Tukey’s test at *p* < 0.05). The capital letters refer to GSSG.

The total glutathione pool was clearly higher in embryonic axes (Figure 2a) than in cotyledons (Figure 2b). The pool decreased, reaching values 6 times lower than those at the beginning (Figure 2a). Total glutathione decreased in cotyledons and was extremely low at the 15–18th WAF range, being 2–4 times lower than the lowest content detected in embryonic axes.

Glutathione was predominantly reported in the reduced form (Figure 2a,b); however, the degree of reduction and oxidation of glutathione shifted in different manners in both seed tissues. *E*_GSSG/2GSH_, a parameter representing the changes in cellular redox homeostasis, indicated highly reducing environment in embryonic axes up to the 16th WAF (Figure 2c). In contrast, the *E*_GSSG/2GSH_ pointed to a more oxidizing cellular environment in cotyledons, particularly at the 15th WAF, when nearly 80% of glutathione was the oxidized form (Figure 2d).

### 3.3. Pyridine Nucleotides

**Figure 3:**
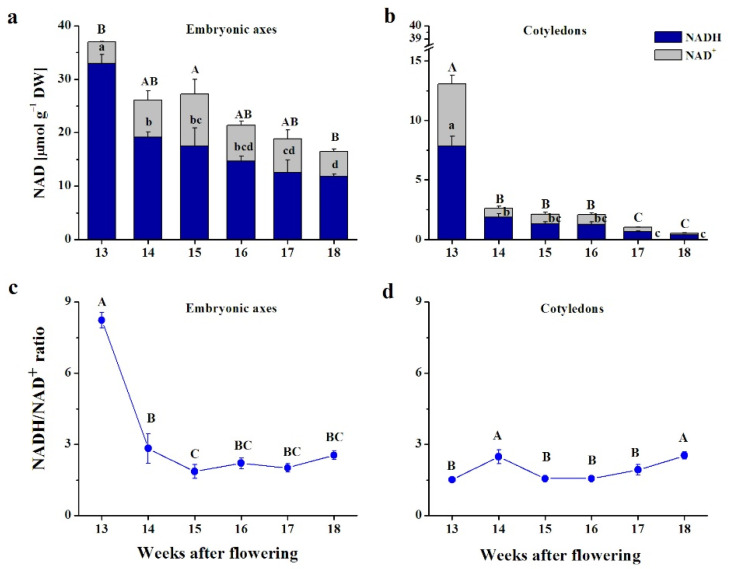
Nicotinamide adenine dinucleotide (NAD) levels (**a**,**b**) and the ratio of NAD reduced to the oxidized form (NADH/NAD^+^) (**c**,**d**) in embryonic axes (**a**,**c**) and cotyledons (**b**,**d**) of developing beech seeds. Data are the means of three independent replicates ± the standard error. Statistically significant differences are indicated with different letters (one-way ANOVA, followed by Tukey’s test at *p* < 0.05). The capital letters refer to NAD^+^.

The NAD content was evidently higher in embryonic axes than in cotyledons (Figure 3a,b). The NAD content gradually decreased in embryonic axes, and was nearly halved at the end of maturation (Figure 3a). The reduced form was predominant, comprising 90% of the NAD pool at the 13th WAF and 70% at the latter stages. In cotyledons, the participation of NADH in the NAD pool was more unified, and constituted 60–70% (Figure 3b). Interestingly, NAD levels dramatically decreased at the early maturation stages and remained 5 times lower at the 14–16th WAF, and further decreased until the end of maturation, being 20 times lower than at the beginning.

The NADH/NAD^+^ ratio was exceptionally high in embryonic axes at the earliest maturation stage (Figure 3c). After that, the ratio decreased and remained at a level between 2 and 3. The NADH/NAD^+^ ratio exhibited different patterns of changes in cotyledons (Figure 3d). The ratio was constant, reaching the highest values at the 14th and 18th WAFs.

**Figure 4:**
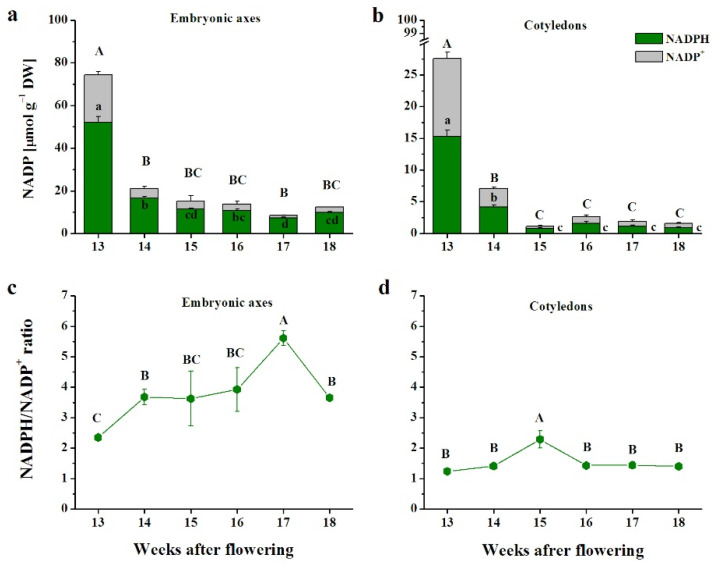
Nicotinamide adenine dinucleotide (NADP) levels (**a**,**b**) and the ratio of NAD reduced to the oxidized form (NADPH/NADP^+^) (**c**,**d**) in embryonic axes (**a**,**c**) and cotyledons (**b**,**d**) of developing beech seeds. Data are the means of three independent replicates ± the standard error. Statistically significant differences are indicated with different letters (one-way ANOVA, followed by Tukey’s test at *p* < 0.05). The capital letters refer to NADP^+^.

Throughout maturation, the NADP content was two times higher in embryonic axes than in cotyledons (Figure 4). The highest content was reported at the 13th WAF, after which five times lower, on average, values were reported (Figure 4a). In cotyledons, the participation of NADH in the NAD pool was unified except at 15 WAF, when NADH peaked and constituted 70% of the pool (Figure 4b). Interestingly, NAD levels dramatically decreased at the early maturation stages and remained at ultralow levels until the end of maturation, when levels were up to 20 times lower.

The NADPH/NADP^+^ ratio increased up to the 17th WAF in embryonic axes, whereas in cotyledons, the ratio exhibited a different pattern of changes and lower values (Figure 4c,d). The ratio was constant, reaching 1.5, except for the 15th WAF, when it increased to 2 (Figure 4d).

**Figure 5:**
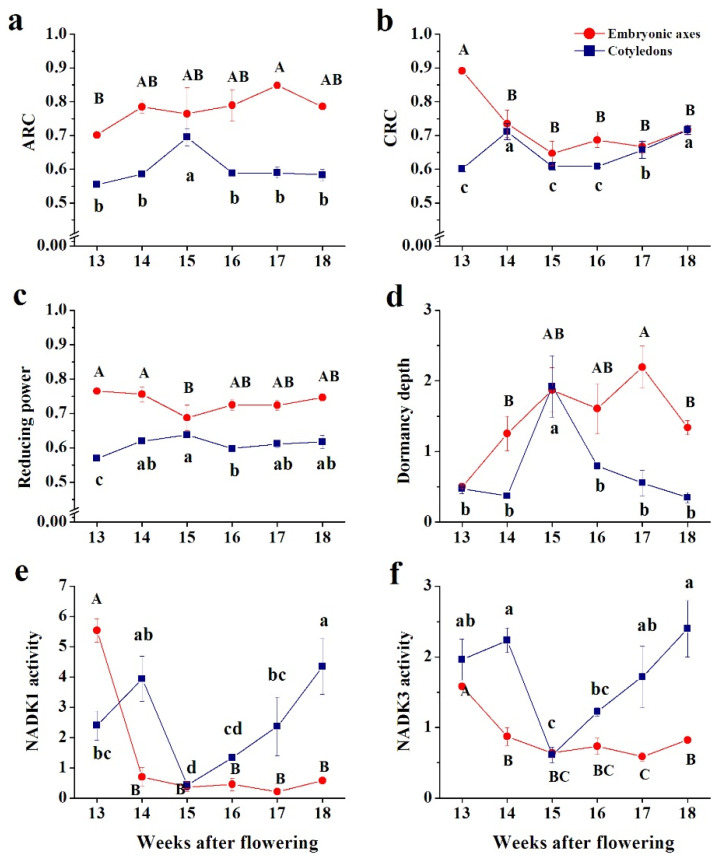
NAD(P)-originated physiological indices: (**a**) metabolism-related catabolic redox charge (CRC) and (**b**) anabolic redox charge (ARC); (**c**) reducing power; (**d**) dormancy depth; phosphorylation capacity of NADK1 (**e**); and NADK3 (**f**) calculated in embryonic axes and cotyledons of developing beech seeds at the 13–18th weeks after flowering (WAF) maturation range. Data represent the mean ± standard deviation of three independent replicates. Statistically significant differences are indicated with different letters (one-way ANOVA followed by Tukey’s test at *p* ≤ 0.05). The capital letters refer to embryonic axes.

NAD(P)-derived indices included metabolism-related parameters. The ARC was substantially higher in embryonic axes and equal between embryonic axes and cotyledons only at the 15th WAF (Figure 5a), which was also reported for other indices (Figure 5c,e,f). CRC was more unified between the embryonic axes and cotyledons throughout the maturation process, except in the initial maturation stage (Figure 5b). Similar to the ARC, the reducing power was definitely higher in embryonic axes, and equaled that of cotyledons solely at the 15th WAF (Figure 5c). The depth of dormancy progressively increased in embryonic axes, and reached the highest and most stable values at the 15–18th WAF range (Figure 5d). The activities of NAD kinases exhibited a similar pattern (Figure 5e,f); however, the activity of NADK1 was at least twice as high as that of NADK3. Interestingly, NAD kinases were more active in cotyledons, except at the 15th WAF stage, when they were identical.

### 3.4. Enzymes Involved in H_2_O_2_ Removal

**Figure 6:**
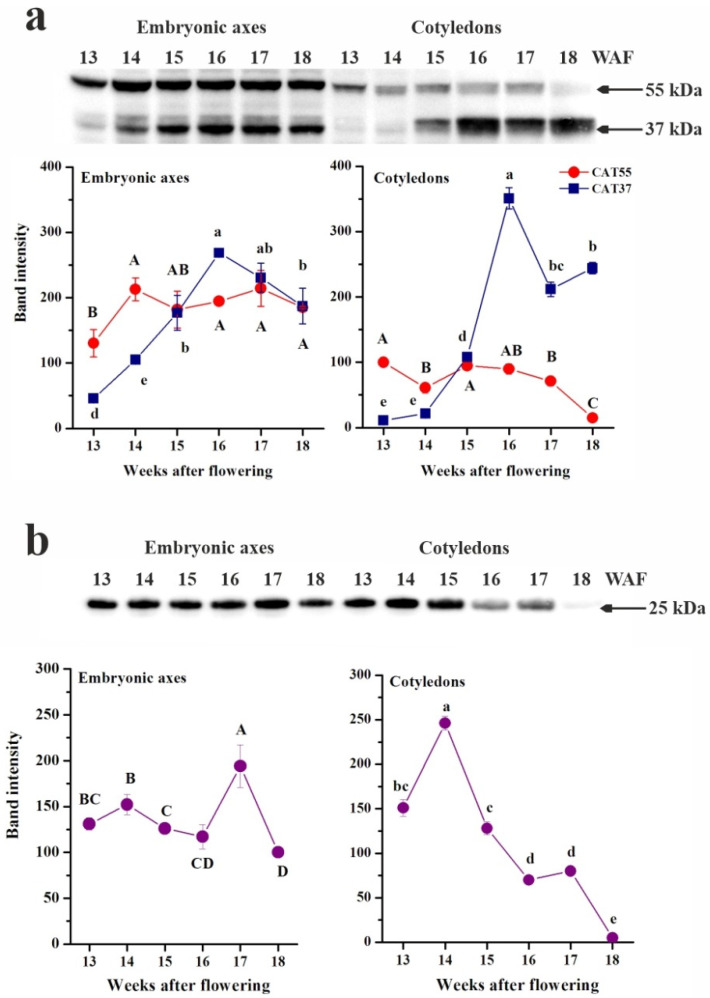
Immunoblot and densitometric analyses of catalase (**a**) and ascorbate peroxidase (**b**) in the embryonic axes and cotyledons of developing beech seeds. Data are shown as the means ± standard deviation (*n* = 3). The same letters indicate groups that are not significantly different according to Tukey’s test.

Similarly to the ascorbate–glutathione cycle, catalase is involved in H_2_O_2_ removal. Two major protein bands, approximately 55 (CAT55) and 37 kDa (CAT37), were recognized by antibodies raised against catalase (Figure 6a). The abundance of the CAT55 protein remained relatively unchanged in embryonic axes throughout seed maturation, whereas in cotyledons, this protein was hardly detectable. Interestingly, the CAT37 protein, which was also more abundant in embryonic axes, clearly responded to maturation-related water loss, and its abundance increased, reaching its highest level at the 16th WAF.

The abundance of the APX in embryonic axes slightly decreased during maturation, except the 17th WAF stage (Figure 6b). Decreasing tendency was more pronounced in cotyledons, particularly at the end of seed development, when this protein was hardly detectable.

### 3.5. Correlations

**Figure 7:**
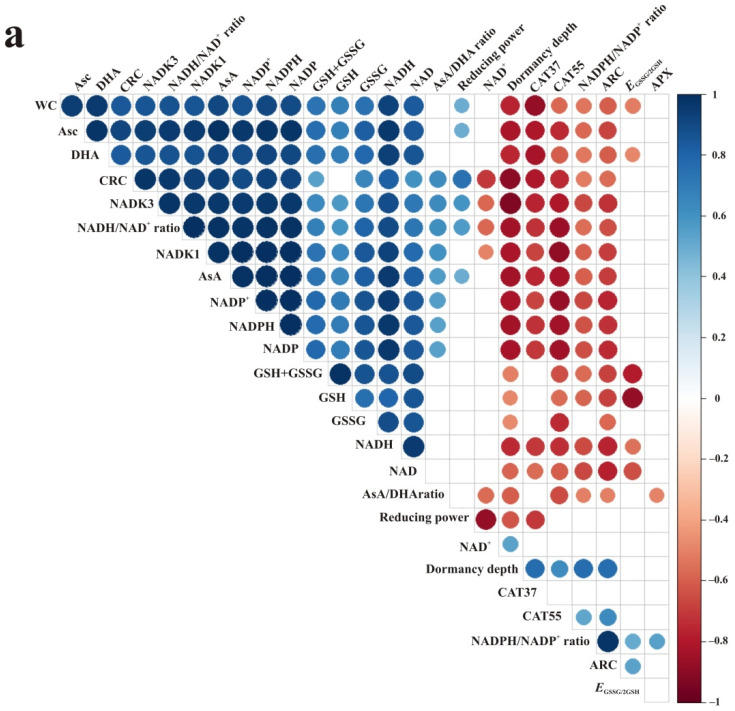

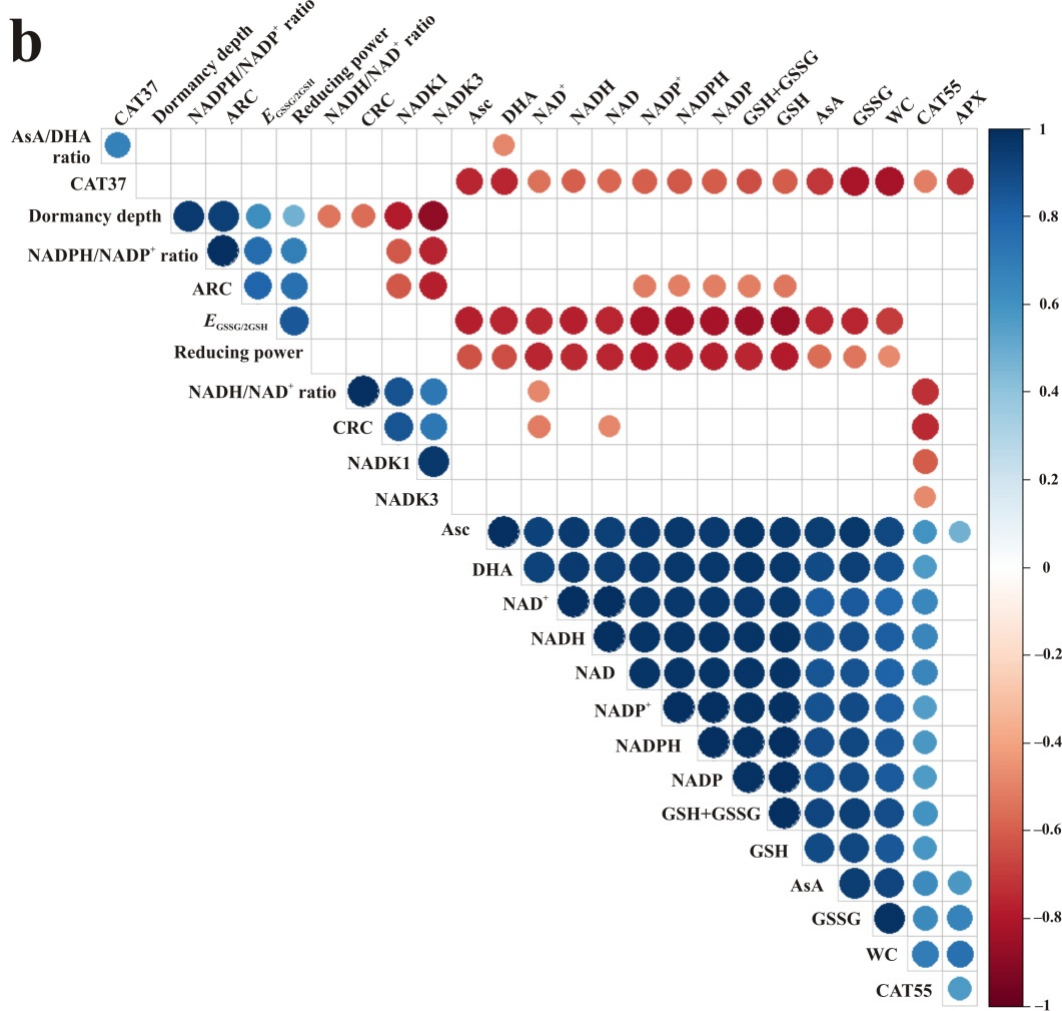
Heatmaps of correlation matrices determined for embryonic axes (**a**) and cotyledons (**b**) of developing beech seeds calculated between levels of ascorbate (Asc), ascorbic acid (AsA), dehydroascorbate (DHA), reduced (GSH) and oxidized (GSSG) glutathione, half-cell reduction potential of glutathione (*E*_GSSG/2GSH_), contents of nicotinamide dinucleotide (NAD) phosphate (NADP) redox forms and their ratios, anabolic redox charge (ARC), catabolic redox charge (CRC), dormancy depth, phosphorylation capacity of isoform 1 (NADK1) and isoform 3 (NADK3) of NAD kinase and reducing power, and abundance of ascorbate peroxidase (APX) and catalases (CAT37, CAT55). Proportional data were arcsine transformed. Color intensity and the size of the circle are proportional to the correlation coefficients. Blanks indicate nonsignificant correlations (*p* > 0.05).

All reported correlations between the content of redox forms of NAD(P) and Asc and glutathione were positive (Figure 7), strongly supporting the hypothesis that NAD(P) are limitation factors in the ascorbate–glutathione cycle. Levels of NADH and NADPH were correlated with reduced and oxidized forms of both ascorbate and glutathione, and these correlations were stronger for ascorbate in embryonic axes and conversely stronger for glutathione in cotyledons. In embryonic axes, ARC was strongly negatively correlated with the content of Asc and glutathione redox forms, while a strong positive correlation was reported between CRC, NADK1, NADK3, and the content of Asc and glutathione redox forms (Figure 7a). Fewer correlations were detected for NAD(P)-derived indices in cotyledons (Figure 7b). Importantly, *E*_GSSG/2GSH_ was positively correlated with the NADPH/NADP^+^ ratio both in embryonic axes and cotyledons, emphasizing that pyridine nucleotides are the driving force of the ascorbate–glutathione cycle, and therefore strongly contribute to the cellular redox homeostasis.

Interesting relationships were revealed for the abundance of CAT proteins. In embryonic axes, the abundance of both CAT55 and CAT37 was negatively correlated with all forms of Asc and NADP and with NADH and CRC, whereas CAT55 was additionally negatively correlated with all forms of glutathione and positively correlated with the NADPH/NADP^+^ ratio and ARC (Figure 7a). In cotyledons, the two detected CATs displayed contrasting correlations (Figure 7b). More precisely, the abundance of CAT55 was positively correlated with all forms of Asc, glutathione, NAD, NADP, and WC, whereas CAT37 exhibited negative correlations with the same parameters. Moreover, the abundance of CATs was apparently modulated via redox status manifested by specific ratios. For example, the abundance of CAT55 was negatively correlated with the NADH/NAD^+^ ratio, while CAT37 was positively correlated with the AsA/DHA ratio, and this was the unique positive correlation of this protein.

## 4. Discussion

Xiao et al. [60] were the first to emphasize the role of NAD and NADP redox forms in redox (oxidative and reductive) stress. Both NAD and NADP (NAD(P)) are antioxidant cofactors regulating cellular redox homeostasis, and at the same time, pro-oxidants inducing redox stress. Thus, redox stress would depend on the redox ratio of each. Deficiency or imbalance of NAD(P) redox forms is associated with many pathological disorders [61]. NAD^+^ is necessary for glycolysis, while NADH provides electrons for ATP production in mitochondria [62]. Both NADH and NADPH administrate reducing power for the functioning of the ascorbate–glutathione cycle in plant organs [30,63,64], including developing seeds [22,56]. Importantly, the correlation between NAD(P) and redox forms of Asc and glutathione was positive (Figure 7), and their redox switches depended on each other. In this context, the availability of NAD(P) might be considered the driving force for the ascorbate–glutathione cycle, determining its efficiency in this study. The ascorbate–glutathione cycle was previously investigated in developing beech seeds, but with no regard to NAD(P) redox forms, and with the use of different and separate methods to detect redox forms of Asc and glutathione [65]. Recently, Jung et al. [66] termed this cycle the AsA-GSH-NADPH cycle, and indicated that AsA applied exogenously to seeds plays a critical role in regulating the homeostasis of this cycle. More specifically, applied AsA increased NADPH levels. The correlation coefficient between AsA and NADPH suggested that the redox ratio of NADP was better regulated in embryonic axes than in cotyledons (Figure 7). This agreed with the fact that at further developmental stages, AsA displays a positive effect on the NADPH redox ratio only in seedling roots but not in leaves [66], and the fact that the general reducing power was much higher in embryonic axes than in cotyledons (Figure 5c). The fact that embryonic axes of nonorthodox seeds had better constitutive antioxidant protection [67] was here supported by the available pools of reduced pyridine nucleotides benefiting embryonic axes.

The average levels of NAD in developing orthodox and recalcitrant seeds were comparable, whereas NADP levels were halved in recalcitrant seeds [22]. Therefore, NADPH is suggested to be the most limiting factor of the ascorbate–glutathione cycle. NAD contents in beech seeds (Figure 3) were lower than those detected in developing seeds of *Acer* [22]. Levels of NADP reported in beech seeds (Figure 4) were comparable to those established in recalcitrant seeds; however, the extremely high NADPH/NADP^+^ ratio, which ranged from 2–5 (Figure 4), was beneficial, particularly for embryonic axes. NADPH is the cofactor of glutathione reductase (GR), and thus, GSH may be the predominant form, displaying up to 5 times higher content than GSSG (Figure 2a,b). On the other hand, NADPH might also act as a pro-oxidant fueling cellular ROS production via NADPH oxidases, known as respiratory burst oxidase homologs (Rbohs). RbohD was identified as a novel negative regulatory gene of seed longevity [68]. In this context, further investigation of Rbohs in beech seeds is crucial to fully understand the role of NADPH. Most likely, the abundance of NADPH contributed to GR activity and GSH regeneration, but the excess GSH did not benefit the AsA pool, most likely due to inefficient DHAR activity, which coincided with the limited longevity of intermediate seeds. This suggestion is in line with the fact that the DHAR, which reconverts DHA into AsA, positively regulates seed longevity [68]. Cell viability is lost faster in cotyledons than in embryonic axes [67]. In this context, the reduced forms of NAD(P) will preferentially stimulate the antioxidant response in the embryonic axes.

The regulation of redox switches in developing seeds is more efficient and dynamic in orthodox than in recalcitrant seeds, as confirmed for the ascorbate–glutathione cycle [33], thiols [18], and Met/MetO [22]. Reduction reactions and regeneration of redoxins are NAD(P)H-dependent [69]. Importantly, NAD(P)-driven redox status contributes to desiccation tolerance [23]. NAD^+^ predominantly accumulates in orthodox-type tissues such as pollen [48] and seeds [23], coinciding with the metabolic switch-off [70]. Beech seeds accumulate the reduced form, NADH, displaying an NADH/NAD^+^ ratio of approximately 2 throughout maturation (Figure 3) and therefore missing this orthodox-type attribute. Therefore, despite the acquisition of desiccation tolerance features [8], beech seeds did not exhibit full orthodox characteristics, and were eventually categorized as intermediate [9]. NAD and NADP modulate multiple key factors in cell death [71], and thus might be involved in the reduced longevity of long-term stored beech seeds. The range of concentrations of the glutathione pool was similar to the range detected in developing *Acer* seeds classified as orthodox [22]; however, a clear descending trend in this characteristic was unique to the developing beech seeds analyzed in this study (Figure 2a). Recalcitrant seeds displayed higher concentrations of Asc than orthodox seeds [72,73]. The accumulation of Asc in recalcitrant *Acer* seeds is a compensation strategy applied to resist oxidative damage [25]. The progressive decline in the content of Asc detected in beech seeds (Figure 1a,b) was in line with previous results [65] and declining patterns of Asc reported in orthodox seeds [22]. Importantly, AsA was found to increase NADPH levels [66]. Therefore, declining levels of AsA probably affected NADPH levels, which was reflected in R^2^ = 1 in embryonic axes (Figure 7a) and R^2^ = 0.88 in cotyledons (Figure 7b), indicating once again that the regulation of the cellular redox status was much more efficient in the embryonic axes than in cotyledons, and pyridine nucleotides contributed notably to this regulation.

NAD kinases (NADKs) phosphorylate NAD to NADP, and their activity is indicated as phosphorylation capacity based on the levels of redox forms of NAD(P) [55]. Several types of NADK exist in Arabidopsis, and include cytosolic (NADK1), chloroplastic (NADK2), and peroxisomal (NADK3) forms [74]. Due to the lack of active chloroplasts in seeds, the activity of the two forms, NADK1 and NADK3, was calculated. The activity of the peroxisomal form was approximately two times higher than that of the cytosolic form, except in the initial maturation stage, when NADK1 was three times more active than NADK3 (Figure 5e,f). Peroxisomal NADPH is involved in NO generation, the β-oxidation pathway, the biosynthesis of jasmonate, and pentose phosphate power [75]. Additionally, NADPH redox power is a connection between several peroxisomal pathways assumed to be important in seed development, seed germination, and postgerminative growth [76]. Exceptionally high NADK1 activity in embryonic axes at the 13th WAF (Figure 5e) resulted in the NADP content exceeding the NAD content (Figure 3 and Figure 4). At this stage, NADP comprised over 66% of the NAD(P) pool, while at later stages, NADP constituted 32–44%. The highest levels of the phosphorylated form of NAD in embryonic axes were in contrast with the lowest levels in cotyledons reported at the 15–16th WAF because at other stages, NADP comprised 80–90% of the NAD(P) pool (Figure 3 and Figure 4).

Substantially low contents of NAD were reported in cotyledons beginning from the 14th WAF, and coincided with the accumulation of storage materials (Figure 3 and Figure S1). Aerobic glycolysis fueled via NAD enables rapid proliferation and cell growth [62]. In this context, the 13th WAF might indicate the last stage of embryogenesis, after which maturation began in developing beech seeds. The NAD/NADP ratio correlated with the depth of dormancy in Arabidopsis seeds [57]. The exact time of acquisition of dormancy in beech seeds has not yet been specified precisely. However, we deduced that dormancy was acquired at the 15th WAF (Figure 5d). NAD(P)H is assumed to be an important signaling molecule in plant development [43,45,57]. The content of NAD(P) redox forms might be transformed to many descriptive physiological indices, including ARC and CRC, which illustrate NAD(P)-driven metabolism and consist of synthesis and degradation reactions, respectively [77]. ARC and CRC were correlated negatively and positively, respectively, with the WC only in embryonic axes (Figure 7a). Differences in seed drying during the late maturation phase (Figure S2) were possibly linked to the reported increasing CRC in cotyledons and relatively unchanged CRC in embryonic axes (Figure 5b). The growth-defense trade-offs are known determinants of the ecology of the plant [78]. The physiological costs (i.e., energy expenditure) of defense might be higher in embryonic axes because respiration, measured via oxygen consumption, and therefore energy production, is much greater in embryonic axes than cotyledons [79].

Native CAT displays heterotetrameric structures in plants with molecular masses of 220–350 kDa and 55–59 kDa subunit sizes [80,81]. According to the UniProt database [82], the *Fagus sylvatica* genome contains two catalases (ID: A0A2N9HF74, A0A2N9HF84) exhibiting molecular masses of 56.7 kDa and 59.5 kDa monomers, similar to Arabidopsis CAT2 and CAT1, respectively, and a smaller catalase domain-containing protein (ID: A0A2N9ECW0) in size (37.5 kDa). In this context, the protein band with higher molecular mass, termed CAT55, most likely refers to CAT2, which was previously reported as characteristic of seeds [83]. CAT was found to act in plant growth and development, including fruit development and ripening of peach [84] and strawberry [85], as well as in the development of sunflower seeds [86], in which CAT was present in glyoxysomes (further transformed to leaf-type peroxisomes) in oil-rich cotyledons [87]. Similarly, beech seeds accumulate oils as up to 40% of their dry mass [88]; however, both detected CAT proteins were more abundant in embryonic axes (Figure 6). The abundance of CATs in developing sunflower seeds was reported to be constant or amplified by dehydration, depending on the isoform [86]. The abundance of both CATs was negatively correlated with WC in embryonic axes, whereas in cotyledons, CAT55 displayed a positive and CAT37 a negative correlation with WC (Figure 7), confirming that only the abundance of CAT55 in cotyledons was not affected by the desiccation associated with maturation. The catalase domain-containing protein displays a full catalase domain, and the major gene ontology annotation for the corresponding gene indicates a role in H_2_O_2_ catabolic process [82]. Therefore, a catalase domain-containing protein, termed CAT37, might contribute to the efficient removal of H_2_O_2_ produced, particularly at the 16th WAF, when desiccation tolerance is acquired [8], supporting the role of the ascorbate–glutathione cycle because the abundances of CATs were correlated with redox forms of Asc, glutathione, NAD, and NADP (Figure 7). More precisely, CAT55 displayed the strongest correlations with the NADPH content, whereas CAT37 was most strongly correlated with the levels of AsA, indicating the interconnections of CAT–AsA–NADPH in the redox network. Although both CAT proteins were strongly related to the content and redox status of NAD(P), it seems that CAT37 will be the major enzyme responsible for H_2_O_2_ removal in cotyledons, particularly at the end of seed development, when the abundance of CAT55 and also APX is negligible.

## 5. Conclusions

NAD(P)H provides reducing power for the ascorbate–glutathione cycle, and redox control of whole plant development has been termed the AsA–GSH–NADPH cycle since last year. Among pyridine nucleotides, NADPH appeared to be the most limiting factor in the cycle efficiency, especially when the NADP pool was comparable to that in developing recalcitrant seeds. Therefore, the lack of NAD^+^ accumulation confirmed that despite the acquisition of desiccation tolerance, the characteristics of developing beech seeds are not fully orthodox. NADPH regenerated GSH via GR, but the AsA/DHA ratio was below 1; therefore, inefficient regeneration of AsA when GSH is available might predefine the reduced storability of beech seeds. In particular, a strong relationship between the contents of AsA and NADPH was revealed, along with the dependency of CAT abundance on AsA and NADPH contents and their ratios.

Reducing power was higher in embryonic axes, indicating that redox homeostasis is better controlled in embryonic axes than cotyledons. Moreover, the contents of redox forms, including Asc, glutathione, NAD, and NADP, were higher long before the accumulation of storage materials and mass maturity in embryonic axes, emphasizing superior protection of this seed tissue. Therefore, further investigations of NAD^+^ synthesis and salvage pathways, together with the activity of NAD(P)-consuming enzymes, particularly in embryonic axes, will allow us to better understand the role of pyridine nucleotides in the regulation of beech seed development apart from their evident role in the regulation of ROS removal via the ascorbate–glutathione cycle and CATs, which was clearly revealed in this study.

## Data Availability

The data is contained within the article.

## Supplementary Materials

The following are available online at https://www.mdpi.com/article/10.3390/antiox10122021/s1, Figure S1: Electrophoresis of proteins, Figure S2: Water content in developing beech seeds.
