# Supplementary material for: NAD(P)H Drives the Ascorbate–Glutathione Cycle and Abundance of Catalase in Developing Beech Seeds Differently in Embryonic Axes and Cotyledons
Source: Antioxidants (Basel). 2021 Dec 20;10(12):2021. doi: 10.3390/antiox10122021 (PMC8698623; doi:10.3390/antiox10122021)
Supplement: Supplementary file 1 [file antioxidants-10-02021-s001.zip › Figure S2.pdf]

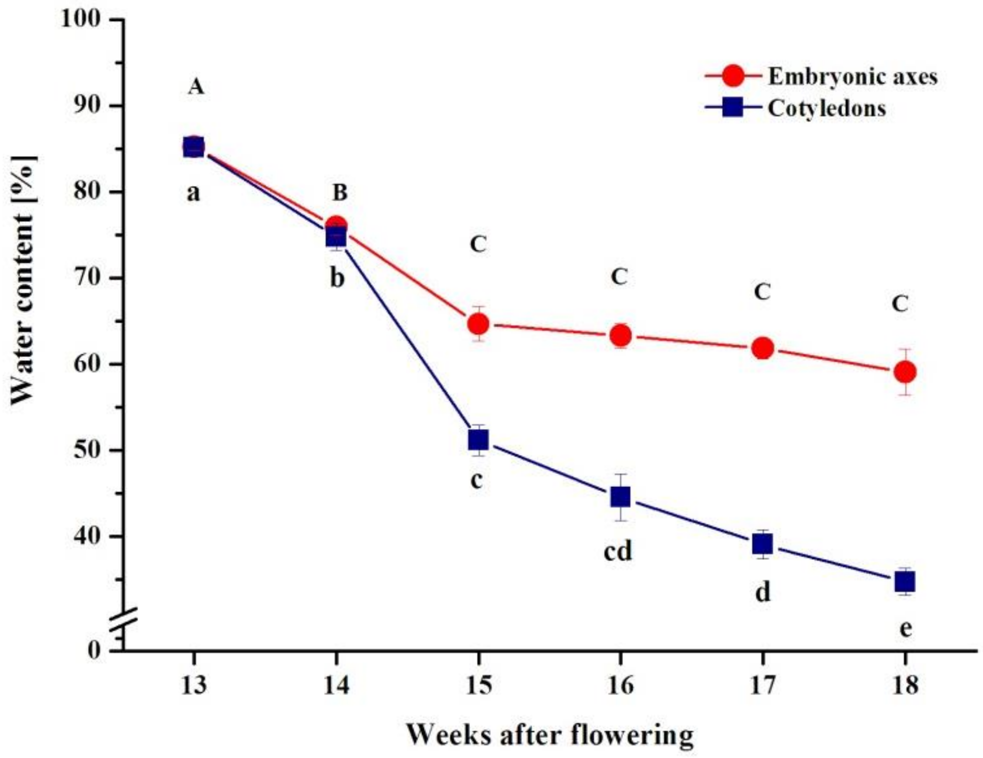

Figure S1. Water content in embryonic axes and cotyledons of developing beech seeds. Data are the means of three independent replicates ± the standard error. Statistically significant differences are indicated with different letters (one-way ANOVA, followed by Tukey's test at $p < 0.05$). The capital letters refer to embryonic axes.
