# Supplementary figures and images for: NAD(P)H Drives the Ascorbate–Glutathione Cycle and Abundance of Catalase in Developing Beech Seeds Differently in Embryonic Axes and Cotyledons
Source: Antioxidants (Basel). 2021 Dec 20;10(12):2021. doi: 10.3390/antiox10122021 (PMC8698623; doi:10.3390/antiox10122021)

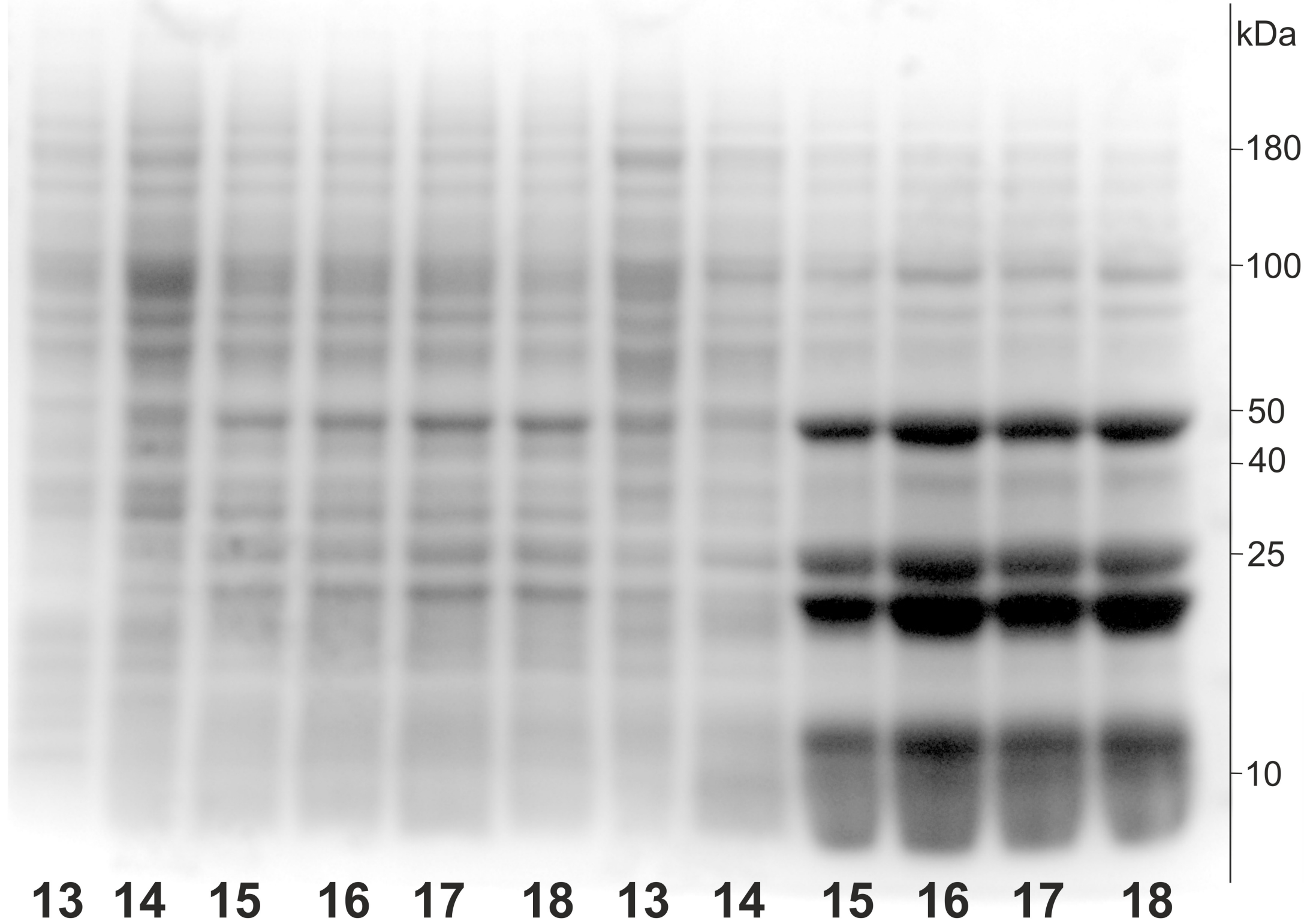

Supplement: Supplementary file 1 [file antioxidants-10-02021-s001.zip › Figure S1.pdf]
